# Cholesterol depletion inhibits BDNF-dependent spike timing-dependent plasticity at thalamo-amygdala synapses

**DOI:** 10.3389/fncel.2026.1769264

**Published:** 2026-02-16

**Authors:** Thomas Munsch, Susanne Meis, Volkmar Lessmann

**Affiliations:** 1 Institute of Physiology, Otto-von-Guericke University, Magdeburg, Germany; 2 Center for Behavioral Brain Sciences, Magdeburg, Germany; 3 DZPG (German Center of Mental Health), partner site Halle/Jena/Magdeburg (CIRC), Magdeburg, Germany

**Keywords:** amygdala, BDNF, lipid rafts, long-term potentiation, LTP, patch-clamp, STDP, TrkB receptor

## Abstract

The neurotrophin brain-derived neurotrophic factor (BDNF) has emerged as a key regulator of synaptic plasticity in hippocampus and cortex of mammalian brains. In the lateral nucleus of the amygdala (LA), BDNF is involved in the control of long-term potentiation (LTP). Here, we show that BDNF is involved in spike-timing dependent potentiation (STDP) of thalamic inputs onto LA projection neurons. Inhibition of BDNF/TrkB signaling with the TrkB scavenger TrkB/FC completely blocked this timing-dependent form of LTP (t-LTP). Disruption of lipid-rafts by depletion of cholesterol from synaptic microdomains with Methyl-β-cyclodextrin (MCD) also prevented induction and expression of t-LTP. These data suggest that BDNF-induced TrkB translocation into synaptic lipid-rafts is required for induction of t-LTP at thalamo-amygdala synapses. Since cholesterol-dependent modulation is not unique for TrkB receptor signaling but has been described for other receptors and ion channels involved in synaptic plasticity, additional studies are required to obtain a more complete picture regarding their role in t-LTP at thalamo-amygdala afferents.

## Introduction

BDNF has been shown to play a key role as a regulator of synaptic strength (Gottmann et al., 2009; Minichiello, 2009; Edelmann et al., 2014; Panja and Bramham, 2014; Park and Poo, 2013). Acting via postsynaptic tropomyosin related receptor kinase B (TrkB), BDNF is involved in inducing long-term potentiation (LTP) in hippocampal and cortical brain slices (see, e.g., Carmignoto et al., 1997; Mohajerani et al., 2007; Andrade-Talavera et al., 2015; Edelmann et al., 2015).

Recently, the relevance of BDNF in induction and expression of LTP has been revealed at thalamic inputs to the lateral amygdala (LA) (Musumeci et al., 2009; Meis et al., 2012, 2020), a nuclear structure which plays a critical role in fear learning (Sigurdsson et al., 2007; Pape and Paré, 2010). Moreover, these thalamic afferents to LA projection neurons also show Hebbian LTP that depends on the timing of action potential firing in presynaptic inputs relative to postsynaptic backpropagating action potentials (bAPs), the so-called spike-timing dependent plasticity (STDP) (Humeau et al., 2005; Shin et al., 2006; Jung et al., 2010). As bAPs are difficult to assess directly in the non-laminated LA, the calcium rise evoked by somatic current injection to generate bAPs was utilized as indirect measure for spike propagation into spines (Humeau et al., 2005).

Importantly, STDP has previously been introduced and thoroughly investigated in hippocampal, cortical and striatal circuits. These studies confirmed the Hebbian learning rule that repeated brief periods of coincident pre- and postsynaptic spiking can either increase or decrease synaptic strength (see, e.g., Markram et al., 1997; Sjöstrom et al., 2008; Bender et al., 2006; Nevian and Sakmann, 2006; Rodrıguez-Moreno and Paulsen, 2008; Rodríguez-Moreno et al., 2011, 2013; Martínez-Gallego et al., 2022; Bi and Poo, 1998; Debanne et al., 1998; Pike et al., 1999; Andrade-Talavera et al., 2016; Falcón-Moya et al., 2020).

However, in the amygdala, STDP mechanisms are thus far less well characterized. STDP at thalamo-LA synapses was shown to be blocked by addition of NMDA receptor antagonists to the ACSF or to the pipette solution (Humeau et al., 2005; Shin et al., 2006; Jung et al., 2010). Moreover, this form of synaptic plasticity was inhibited by postsynaptic perfusion with the Ca^2+^ chelator BAPTA and relied on activation of voltage-dependent R-Type Ca^2+^-channels by bAPs depolarizing large spines contacted by thalamic afferents (Humeau et al., 2005; Shin et al., 2006). Furthermore, STDP expression did not affect the paired pulse ratio of EPSPs during repetitive stimulation (Humeau et al., 2005). These results thereby demonstrated that induction of STDP at thalamic afferents to the LA depends on postsynaptic activation of NMDA receptors and expression of STDP in this circuit relies on postsynaptic signaling cascades.

Interestingly, synaptic plasticity induced at the same thalamic synapses to the LA with another induction protocol relied on similar mechanisms. Thus, we previously showed that LTP induced by high-frequency stimulation of thalamic inputs paired with postsynaptic depolarization depends on postsynaptic NMDA receptor activation and GluR1 insertion (Meis et al., 2012). In addition, this study demonstrated a critical role for postsynaptic BDNF/TrkB signaling in the LTP induction/expression process (Meis et al., 2012). In summary, these previous studies by Humeau et al. (2005) and our own studies demonstrated a conserved mechanism of induction and expression via postsynaptic mechanisms across different LTP induction paradigms, while our own studies indicated a critical role for BDNF signaling for LTP at this thalamic input to the LA. However, whether the STDP burst protocol introduced by Humeau and coworkers is also BDNF-dependent has not been previously investigated.

The effect of mature BDNF on synaptic plasticity mechanisms is mediated by activation of tropomyosin-related kinase B (TrkB) receptors (Gottmann et al., 2009; Minichiello, 2009) and downstream activation of the phosphatidylinositol 3-kinase (PI3K)–Akt pathway, the ERK–MAPK pathway acting via Shc/FRS-2 binding, and the phospholipase C-γ (PLC-γ) pathway (Musumeci et al., 2009; Yoshii and Constantine-Paton, 2010). TrkB receptors and other transmembrane receptors and ion channels are known to be localized in specialized membrane domains enriched in sphingolipids and cholesterol (Karnovsky et al., 1982; for a recent review see Incontro et al., 2025), the so-called lipid rafts, where they are clustered to enable efficient activation of their downstream intracellular effectors i.e.: (Suzuki et al., 2004, Pereira and Chao, 2007, reviewed by Zonta and Minichiello, 2013). In cultured cortical neurons, BDNF binding to TrkB receptors induces recruitment of TrkB receptors into cholesterol-rich lipid rafts (Suzuki et al., 2004). Interestingly, disruption of lipid rafts by cholesterol depletion has been shown to abolish BDNF-dependent potentiation of evoked glutamatergic synaptic responses in cultured cortical neurons and of tetanic stimulation-induced LTP in acute hippocampal slices (Suzuki et al., 2004). However, BDNF-dependent and lipid raft requiring LTP has not previously been shown in amygdala circuits.

In the present study, we first set out to investigate whether the local, afferent-specific t-LTP of thalamic glutamatergic inputs to LA projection neurons introduced by Humeau et al. (2005) depends on BDNF signaling. Secondly, we determined whether recruitment of activated TrkB receptors into postsynaptic lipid raft domains of LA projection neurons is an essential step for this form of t-LTP to occur. To this aim we performed whole-cell patch clamp recordings of LA projection neurons in acutely isolated amygdala slices from juvenile mice. Slices were pre-incubated and superfused throughout recording either with TrkB receptor bodies (TrkB-Fc) to scavenge endogenously released BDNF or with Methyl-β-cyclodextrin (MCD) to inhibit lipid raft interaction (Simons and Toomre, 2000) with TrkB-receptors. Our results show that induction of t-LTP was prevented both by application of TrkB-Fc and also by incubation with MCD.

## Methods

### Experiments were carried out in accordance with the European committees council directive (86/609/EEC)

#### Slice preparation

Coronal slices (300 μm thick) were prepared from 4- to 8-week old C57Bl/6 J mice. Mice were deeply anaesthetized by inhalation of isoflurane (1-chloro-2,2,2-trifluoroethyl-difluoromethylether) and killed by decapitation. A block of tissue containing the amygdala was rapidly removed and placed in chilled oxygenated physiological saline containing (in mM): KCl, 2.4; MgSO_4_, 10; CaCl_2_, 0.5; NaHCO_3_, 24; NaH_2_PO_4_, 1.25; glucose, 10; sucrose, 195; bubbled with 95% O_2_-5% CO_2_. Slices were prepared on a vibrating blade microtome (Model 1,000, The Vibratome Company, St. Louis, MO, USA), and were incubated in standard artificial cerebrospinal fluid (ACSF) of the following composition (in mM): NaCl, 119; KCl, 2.5; NaH_2_PO_4_, 1.25; NaHCO_3_, 25; MgSO_4_, 1; CaCl_2_, 2; glucose, 20; bubbled with 95% O_2_-5% CO_2_. Slices were kept at 32 °C for 20 min followed by incubation at room temperature.

For experiments, single slices were transferred to the recording chamber and submerged in ACSF recirculated in a modified Delta T perfusion system (Bioptechs, Butler, PA, USA) at a rate of 2 mL/min at 32 °C.

#### Whole-cell recordings

Whole-cell recordings of LA projection neurons were obtained with a patch-clamp amplifier (EPC-9, HEKA, Lambrecht, Germany) under visual inspection of slices with DIC infrared illumination through videomicroscopy (CF8/1, Kappa, Gleichen, Germany). Patch pipettes were pulled from borosilicate glass (GC150TF-10, Clark Electromedical Instruments, Pangbourne, UK) to resistances of 3–4 MΩ when filled with a pipette solution containing (in mM): K-gluconate, 135; KCl, 5; Hepes, 10; MgCl_2_, 2; EGTA, 0.2; MgATP, 4; Na_3_-GTP, 0.4; K_3_-phosphocreatine, 10; pH 7.2 with KOH. The liquid junction potential of 10 mV resulting from contact of the intracellular solution with the recording ACSF at the pipette tip was corrected for. LA neurons were routinely held at −70 mV membrane potential, unless indicated otherwise.

**Figure 1:**
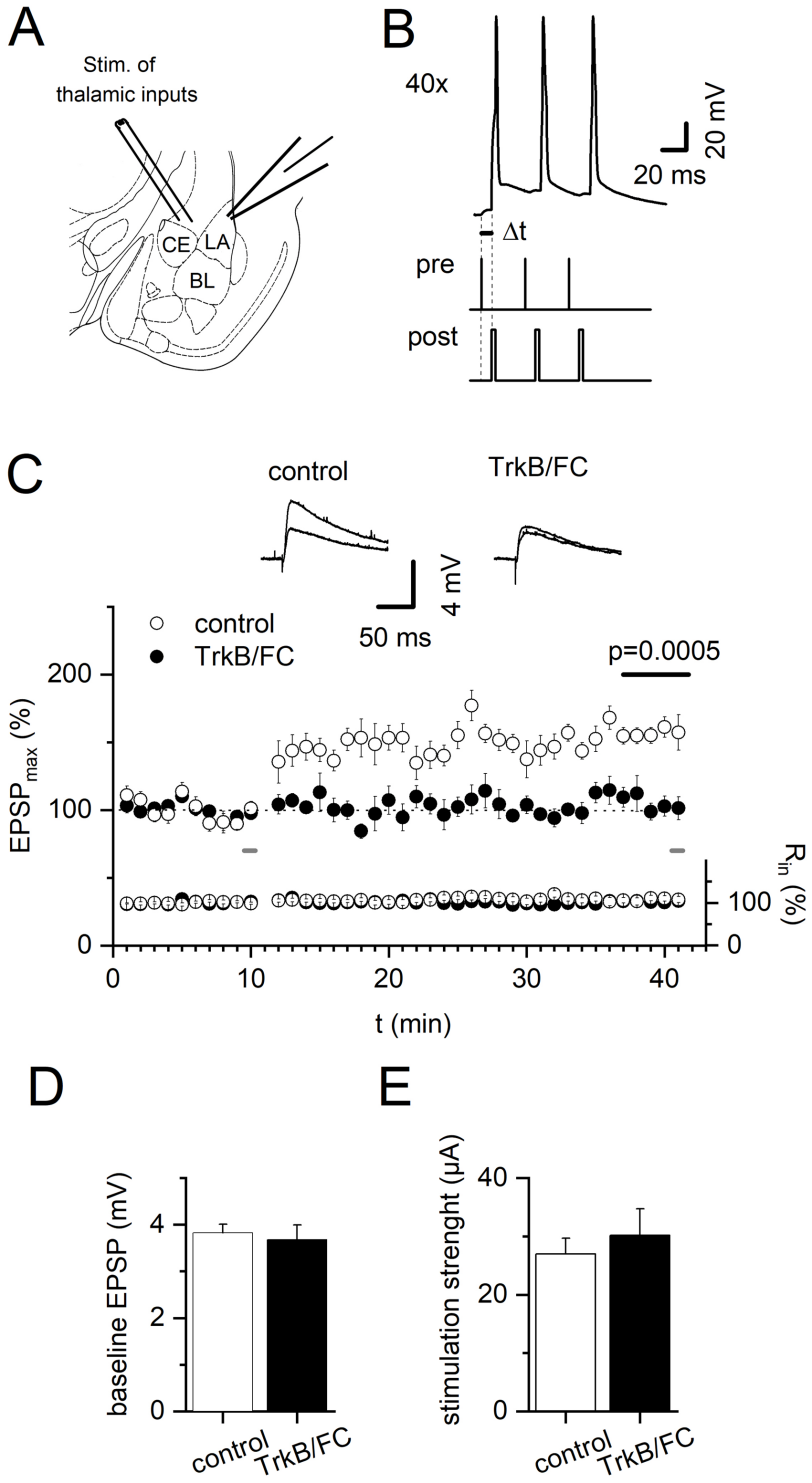
Induction of timing-dependent LTP at thalamo-amygdala synapses depends on acute TrkB signaling. **(A)** Coronal brain slice with the stimulating electrode positioned dorsal to the central amygdala (CE) and the recording electrode in the dorsal part of the lateral amygdala (LA). **(B)** t-LTP was induced by 40 repetitions of pairing 3 EPSPs upon afferent stimulation with 3 backpropagating APs upon depolarizing current injection into postsynaptic neurons. **(C)** Time course of averaged evoked EPSPs and input resistance in response to stimulation of thalamic afferents under control conditions and in the presence of TrkB/FC. Insets show 5 averaged EPSPs before t-LTP induction and at the end of recordings for control and TrkB/FC treated slices, respectively, at indicated times. **(D,E)** Stimulus intensity was adjusted to produce synaptic responses of 2–5 mV. Neither amplitude of the baseline EPSP **(D)** nor stimulation strength **(E)** was different between groups. Control: *n* = 10, TrkB/FC: *n* = 6, *p* = 0.0005.

Excitatory postsynaptic potentials (EPSPs) or currents (EPSCs) were evoked by stimuli of 100 μs duration delivered by a stimulus isolator (Isoflex, AMPI, Jerusalem, Israel) at 0.1 Hz. A connected concentric bipolar electrode (FHC Inc., Bowdoin, ME, USA) was placed on the slice surface dorsal to the central nucleus of the amygdala for stimulating the thalamic input to the LA (Figure 1A). Stimulus intensity was adjusted to elicit synaptic responses with amplitudes of 2–5 mV (when EPSPs were recorded in current clamp) or 100–150 pA (when EPSCs were recorded in voltage clamp). All recordings were performed in the presence of the GABA_A_ receptor antagonist Gabazine (10 μM) and the GABA_B_ receptor antagonist CGP55845 (10 μM) in the ACSF. Peak amplitudes of EPSPs and EPSCs were calculated by averaging four consecutive responses. Timing-dependent LTP (t-LTP) was induced by pairing 40 bursts (intraburst frequency = 30 Hz, interburst frequency = 0.2 Hz) of 3 EPSPs and 3 backpropagating APs (Δt: 10 ms) elicited by 2 ms injections of −1 nA into the postsynaptic neuron (EPSP-bAP delay of +5 to +10 ms, Figure 1B; Humeau et al., 2005). t-LTP was quantified by normalizing and averaging peak EPSP amplitudes during the last 5 min of experiments relative to the 5 min of baseline immediately before the t-LTP induction protocol. In experiments where we applied the soluble BDNF scavenger human recombinant TrkB/FC (Shelton et al., 1995; Walz et al., 2006), slices were preincubated in an interface chamber for 40 to 45 min with TrkB/FC (2 μg/mL ACSF). In addition, the same concentration of TrkB/FC was present throughout the experiment in the recording chamber. Interleaved control recordings in separate slices were treated identically but without addition of TrkB/FC to the ACSF.

In experiments using Methyl-β-cyclodextrin (MCD), slices were also preincubated in an interface chamber for at least 30 min in the presence of MCD (2 mM). MCD raise the solubility of cholesterol by incorporating it into its hydrophobic cavity and forming inclusion complexes (Eckert, 2010). In addition, ACSF was supplemented during recordings with the same concentration of MCD. In some control experiments, MCD (2 mM) was added after 10 min of baseline EPSC recording without induction of t-LTP, and peak EPSP amplitudes during the last 5 min in the presence of MCD were quantified relative to the 5 min baseline just before addition of MCD.

#### Data analysis

Data were analyzed with Origin 8.0 (OriginLab Corp., Northampton, MA, USA). Statistical analysis was performed using non-parametric tests (Wilcoxon’s signed rank test for paired observations and Mann–Whitney’s test for non-paired observations) by GraphPad Prism software (GraphPad Software, San Diego, CA, USA). Data are presented as means ± SEM. Differences were considered statistically significant at *p* ≤ 0.05.

## Results

Whole-cell recordings were obtained from pyramidal-shaped neurons in the dorsal subdivision of the LA (Meis et al., 2008). In the presence of the GABA_A_ receptor antagonists Gabazine (10 μM) and the presence of the GABA_B_ receptor antagonist CGP 55845 (10 μM), 40 repetitions of pairing of 3 EPSPs elicited by afferent stimulation, with 3 backpropagating action potentials (bAPs) provoked by depolarizing current injection into the postsynaptic neuron induced t-LTP at thalamo-amygdala synapses. The average EPSP amplitude significantly (*p* < 0.05) increased 30 min after the induction protocol to 156.5 ± 5.77% of baseline levels (*n* = 10, Figure 1C). High frequency stimulation (HFS) paired with postsynaptic depolarization at the same thalamic inputs to the LA as investigated here was shown previously to depend on NMDAR activation and BDNF signaling (Meis et al., 2012). Therefore, we next tested whether the burst firing induced t-LTP also depends on acute TrkB signaling. To this aim, we incubated slices with the BDNF-scavenger TrkB/FC (2 μg/mL ACSF) that binds endogenously released BDNF thereby hindering its binding to cellular TrkB receptors (compare Walz et al., 2006). This incubation started at least 30 min before recording and continued during the entire recording period. Treatment of slices with TrkB/FC resulted in complete loss of timing-dependent potentiation (104.9 ± 5.6%, *n* = 6, Figure 1C) when compared to interleaved controls (*p* = 0.0005, Figure 1C). The amplitude of the baseline EPSP (control: 3.83 ± 0.18 mV, *n* = 10; TrkB/FC: 3.68 ± 0.32 mV, *n* = 6; *p* = 0.4923; Figure 1D) and the stimulation strength (control: 27.0 ± 2.7 μA, *n* = 10; TrkB/FC: 30.17 ± 4.6, *n* = 6; *p* = 0.6168; Figure 1E) were not different between the two groups. This indicates that basal synaptic transmission was not altered by chronic BDNF depletion through scavenging with TrkB/Fc.

**Figure 2:**
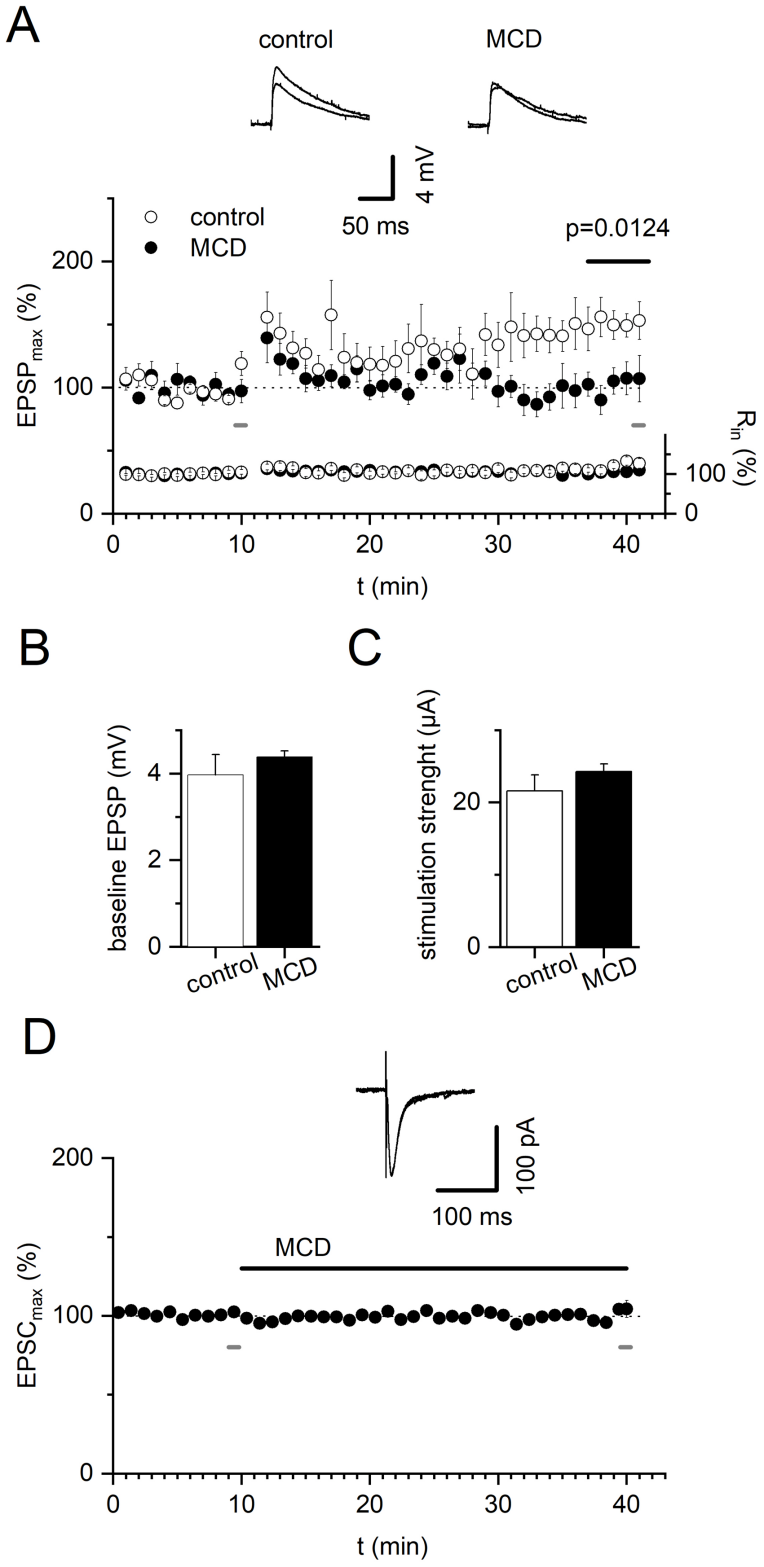
Impairment of timing-dependent LTP at thalamic inputs to the LA by lipid raft disruption with Methyl-β-cyclodextrin. Timing-dependent LTP was induced by 40 repetitions of pairing 3 EPSPs induced by afferent stimulation with 3 bAPs upon depolarizing current injection into postsynaptic neurons. **(A)** Time course of averaged evoked EPSPs and input resistance in response to stimulation of thalamic afferents under control conditions and in the presence of MCD (2 mM) throughout the experiment. Insets show 5 averaged EPSPs before t-LTP induction and at the end of recordings for control and MCD treated slices, respectively, at indicated times. **(B,C)** Stimulus intensity was adjusted to elicit synaptic responses of 2–5 mV. Neither amplitude of the baseline EPSP. **(B)** nor stimulation strength. **(C)** was different between groups. Control: *n* = 6, MCD: *n* = 5, *p* = 0.0124. **(D)** MCD effect on baseline EPSCs. Baseline EPSCs in response to stimulation of thalamic afferents were not affected by addition of MCD (2 mM) after 10 min. Inset shows 5 averaged EPSCs before addition of MCD and at the end of recordings in the presence of MCD at indicated times. *n* = 5, *p* > 0.9999.

Translocation of activated TrkB receptors into synaptic lipid rafts has been proposed to underlie BDNF-dependent synaptic plasticity in CNS neurons (Suzuki et al., 2004). To test whether lipid raft disruption may prevent t-LTP at thalamo-amygdala synapses, we incubated slices with MCD (2 mM) at least 30 min before and throughout the recording. Treatment of slices with MCD again resulted in complete lack of timing-dependent potentiation (100.4 ± 10.71%, *n* = 6, Figure 2A) when compared to interleaved negative control recordings of t-LTP in the absence of MCD (150.8 ± 12.20%, *n* = 5, *p* = 0.0124; Figure 2A). The amplitude of baseline EPSPs (control: 3.96 ± 0.47 mV, *n* = 5; MCD: 4.38 ± 0.14 mV; *n* = 6; *p* = 0.329; Figure 2B) and the required stimulation strength to elicit these responses (control: 21.60 ± 2.21 μA, *n* = 5; MCD: 24.25 ± 1.06 μA, *n* = 6; *p* = 0.355; Figure 2C) were not different between the two groups. Thus, MCD application did not change basal synaptic transmission under our recording conditions.

Recently, cholesterol modulation of basal AMPA receptor dependent excitatory synaptic transmission in autaptic hippocampal cultures by MCD was reported (Korinek et al., 2020). As a further control experiment, we therefore investigated the effect of acute MCD application on evoked EPSCs recorded in amygdala slices upon stimulation of thalamic inputs to LA principal neurons without induction of t-LTP. MCD (2 mM) added after 10 min of baseline recording for 30 min did not lead to significant changes in the average EPSC amplitudes during the last 5 min of experiments relative to 10 min of baseline recording (baseline: 100.3 ± 0.68 pA, MCD: 99.3 ± 1.6 pA; *n* = 5, *p* > 0.9999; Figure 2C).

## Discussion

In the present study we demonstrate that induction of timing-dependent LTP (t-LTP) at thalamo-amygdala synapses depends on activation of acute TrkB signaling through endogenously released BDNF. Moreover, our results show that disruption of synaptic lipid rafts with MCD completely abolished t-LTP suggesting that TrkB signaling through lipid rafts is essential for t-LTP induced by burst-firing STDP protocols. These results indicate that postsynaptic BDNF/TrkB signaling is critically involved in induction and expression of t-LTP at thalamo-amygdala inputs.

BDNF has been found to play a major role in the control of synaptic transmission and activity-dependent synaptic plasticity (Gottmann et al., 2009; Minichiello, 2009; Park and Poo, 2013; Edelmann et al., 2014). At thalamic inputs to the LA, LTP induced by pairing high frequency stimulation (HFS) with postsynaptic depolarization, was completely blocked in the presence of the BDNF scavenger TrkB-FC or inclusion of the Trk receptor antagonist K252a in the pipette solution, respectively (Meis et al., 2012). These results point to a critical role of postsynaptic BDNF/TrkB signaling in the LTP induction/expression process at these synapses (Meis et al., 2012). Likewise, t-LTP at the same synapses that were also investigated in the present study, was absent when the action of endogenously released BDNF during time-dependent pairing of presynaptic inputs and postsynaptic APs was blocked by scavenging of released BDNF (compare Walz et al., 2006). There exist several lines of evidence that both types of LTP (i.e., HFS-dependent LTP and t-LTP) at thalamo-amygdala synapses are expressed at postsynaptic sites via insertion of GluR1-containing AMPA receptors (Humeau et al., 2005, 2007; Rumpel et al., 2005; Meis et al., 2012). This suggests that postsynaptic TrkB signaling is responsible for insertion of GluR1-containing AMPA receptors. An identical mechanism of action has been proven previously for theta burst firing induced t-LTP at Schaffer collateral CA1 synapses in mouse hippocampal slices (Edelmann et al., 2015; Harward et al., 2016). Together, these findings provide evidence that BDNF/TrkB signaling regulated t-LTP is a feature of glutamatergic synapses stimulated by postsynaptic burst firing.

There is increasing evidence that TrkB receptors and their downstream effectors are organized in synaptic lipid raft microdomains (Suzuki et al., 2004; Pereira and Chao, 2007). It is assumed that upon activation by BDNF, TrkB receptors are rapidly translocated from extrasynaptic sites into sub-synaptic lipid rafts where it may activate the downstream signaling cascades (Nagappan and Lu, 2005; Zonta and Minichiello, 2013). Recently, such TrkB receptor movement into synaptic lipid rafts was demonstrated to underlie acute BDNF mediated effects in cultured cortical neurons (Suzuki et al., 2004). Moreover, disruption of lipid rafts by MCD, blocked BDNF-induced TrkB translocation and prevented synaptic plasticity in response to tetanic stimulation in hippocampal slice preparations (Suzuki et al., 2004). In the present study, induction of t-LTP upon stimulation of endogenous BDNF release could also be blocked by disruption of lipid rafts in the presence of MCD. Thus, our present study extends these previous results by showing that endogenous BDNF released with physiologically relevant t-LTP paradigms requires TrkB signaling through postsynaptic lipid rafts.

Regarding modulation of basal excitatory synaptic transmission and LTP induction upon cholesterol depletion protocols with MCD, contrasting findings were reported. These differences most likely reflect distinct experimental conditions (e.g., species, MCD application method, degree of cholesterol depletion, LTP induction protocol, extra-vs. intracellular recording of synaptic responses) between studies. In fact, cholesterol depletion by treatment with MCD was shown to strongly reduce basal synaptic transmission and concurrently inhibit expression of LTP in acute rat hippocampal slices (Koudinov and Koudinova, 2001; Frank et al., 2008; Choi et al., 2015). LTP was also significantly diminished in the presence of MCD in hippocampal slices prepared from guinea pigs (Maggo and Ashton, 2014). In contrast, cholesterol removal by MCD led to enhanced excitatory synaptic transmission and concordantly increased NMDA receptor-dependent LTP in organotypic hippocampal slice cultures (Brachet et al., 2015). However, in acute amygdala slices from mice, as demonstrated in the present study (compare Figures 2B–D), basal synaptic transmission, i.e., evoked EPCSs, were not affected by MCD, whereas BDNF-dependent t-LTP was blocked.

There is evidence that cholesterol-rich lipid raft membrane domains are also involved in control of AMPA receptor movement and/or insertion of AMPA receptors into postsynaptic membranes upon induction of synaptic plasticity (Korinek et al., 2020). Since the induction and expression of t-LTP in the LA depends on AMPA receptor insertion (Humeau et al., 2007), we cannot rule out completely that the effects of MCD on t-LTP observed in the present study also involve inhibition of AMPA receptor insertion. However, the studies mentioned above showed MCD induced modification of basal synaptic transmission and LTP in the same direction, e.g., both were enhanced (Brachet et al., 2015) or reduced (Koudinov and Koudinova, 2001; Frank et al., 2008; Choi et al., 2015). As in our present study, evoked EPCSs were unaffected by MCD application while t-LTP was blocked in the presence of MCD, we propose that the main mechanism of MCD action in our experiments relies on the prevention of TrkB receptor translocation into subsynaptic lipid rafts.

Of note, cholesterol modulation of membrane proteins is not unique for TrkB receptors and many other receptors and ion channels involved in t-LTP induction or expression can be modulated by cholesterol depletion in the cell membrane (for a recent review see Incontro et al., 2025). Interestingly, activation of R-type voltage-dependent Ca^2+^ channels preferentially located at thalamic inputs to the LA was mandatory for the occurrence of t-LTP at this input (Humeau et al., 2005). In cerebellar neurons, cholesterol depletion enhances calcium currents (Davies et al., 2006; Incontro et al., 2025). Given that Ca^2+^ influx is a strong induction signal for LTP and LTD at glutamatergic synapses, such an increase would be expected to affect synaptic plasticity. In the present study, we observed inhibition of t-LTP by cholesterol depletion, but neither enhanced nor reduced basal synaptic transmission during 45 min of MCD application. We therefore conclude that in our preparation, effects of cholesterol on calcium channels do not play a major role. Furthermore, cholesterol was hypothesized to regulate action potential propagation at autapses of hippocampal neurons in cell culture (Korinek et al., 2020; Incontro et al., 2025). Reduction in the amplitude of bAPs could affect thalamic afferents that synapse on distal dendritic processes (Woodson et al., 2000). In addition, cholesterol depletion diminished NMDA receptor responses in cultured rat cerebellar granule cells (Korinek et al., 2015). As t-LTP at thalamic afferents to the LA is blocked by a competitive NMDA receptor antagonist (Humeau et al., 2005), reduction of NMDA receptor currents by cholesterol depletion could reduce t-LTP in the present study. If these processes are encountered in our amygdala preparation is currently unclear and has to be clarified in future studies.

BDNF/TrkB signaling in amygdala circuits contributes critically to cued fear learning. Inhibition of BDNF/TrkB signaling in this brain structure impaired fear memory as well as LTP at thalamic and cortical afferents to the LA (for review and references, see Meis et al., 2020). Importantly, cholesterol plays a complex role in synaptic function as well as learning and memory (Schreurs, 2010; Zhang et al., 2026). It was recently shown that TrkB receptors have a cholesterol-sensing ability that is very likely involved in behavioral and synaptic plasticity effects of BDNF/TrkB signaling (Casarotto et al., 2021). These findings are consistent with our finding that TrkB translocation into synaptic lipid-rafts is required for t-LTP induction in the LA, and may play a significant role in amygdala related learning processes. In addition, dysregulation of cholesterol metabolism may play a pivotal role in neurodegenerative diseases (Zhang et al., 2026). Indeed, the amygdala plays a central role in neurological dysfunctions (Nikolenko et al., 2020), and thus the complex interaction between cholesterol and TrkB signaling may contribute to amygdala-dependent pathophysiology.

Interestingly, in amygdala-rich brain samples, a significant reduction in cholesterol content was observed in mice fed with a high ω3 to ω6 polyunsaturated fatty acid (PUFA) ratio diet compared to mice fed with a low ω3 to ω6 PUFA ratio diet. Importantly, these mice showed less LTP at cortico-LA afferents. In parallel, auditory fear responses were attenuated (Yamada et al., 2016). Overall this suggests important physiological consequences when cholesterol and lipid constitution of cell membranes is altered in amygdala circuits.

Taken together, the presence or depletion of cholesterol and other lipids can affect the function of many different receptors and ion channels in the cell membrane. We provide some control experiments that speak against MCD effects on presynaptic ion channels and membrane proteins involved in glutamate release, and against changes in postsynaptic function of AMPA receptors. However, we cannot exclude contribution of cholesterol-dependent effects—other than inhibited TrkB transfer into subsynaptic lipid rafts – in the presence of MCD in our t-LTP recordings. Further studies will be required to more thoroughly analyse the impact of lipid metabolism in synaptic plasticity in the amygdala and other brain regions and its relation to learning and memory processes.

## Data Availability

The raw data supporting the conclusions of this article will be made available by the corresponding author upon reasonable request.

## References

[ref1] Andrade-Talavera Y. Benito I. Casañas J. J. Rodríguez-Moreno A. Montesinos M. L. (2015). Rapamycin restores BDNF-LTP and the persistence of long-term memory in a model of Down’s syndrome. Neurobiol. Dis. 82, 516–525. doi: 10.1016/j.nbd.2015.09.005 26388397

[ref2] Andrade-Talavera Y. Duque-Feria P. Paulsen O. Rodríguez-Moreno A. (2016). Presynaptic spike timing-dependent long-term depression in the mouse Hippocampus. Cereb. Cortex 26, 3637–3654. doi: 10.1093/cercor/bhw172, 27282393 PMC4961031

[ref3] Bender V. A. Bender K. J. Brasier D. J. Feldman D. E. (2006). Two coincidence detectors for spike timing-dependent plasticity in somatosensory cortex. J. Neurosci. 26, 4166–4177. doi: 10.1523/JNEUROSCI.0176-06.2006, 16624937 PMC3071735

[ref4] Bi G. Q. Poo M. M. (1998). Synaptic modifications in cultured hippocampal neurons: dependence on spike timing, synaptic strength, and postsynaptic cell type. J. Neurosci. 18, 10464–10472. doi: 10.1523/JNEUROSCI.18-24-10464.1998, 9852584 PMC6793365

[ref5] Brachet A. Norwood S. Brouwers J. F. Palomer E. Helms J. B. Dotti C. G. . (2015). LTP-triggered cholesterol redistribution activates Cdc42 and drives AMPA receptor synaptic delivery. J. Cell Biol. 208, 791–806. doi: 10.1083/jcb.201407122, 25753037 PMC4362467

[ref6] Carmignoto G. Pizzorusso T. Tia S. Vicini S. (1997). Brain-derived neurotrophic factor and nerve growth factor potentiate excitatory synaptic transmission in the rat visual cortex. J. Physiol. 498, 153–164. doi: 10.1113/jphysiol.1997.sp021848 9023775 PMC1159241

[ref7] Casarotto P. C. Girych M. Fred S. M. Kovaleva V. Moliner R. Enkavi G. . (2021). Antidepressant drugs act by directly binding to TRKB neurotrophin receptors. Cell 184, 1299–1313.e19. doi: 10.1016/j.cell.2021.01.034, 33606976 PMC7938888

[ref8] Choi T. Y. Jung S. Nah J. Ko H. Y. Jo S. H. Chung G. . (2015). Low levels of methyl beta-cyclodextrin disrupt GluA1-dependent synaptic potentiation but not synaptic depression. J. Neurochem. 132, 276–285. doi: 10.1111/jnc.12995, 25418874

[ref9] Davies A. Douglas L. Hendrich J. Wratten J. Tran Van Minh A. Foucault I. . (2006). The calcium channel alpha2delta-2 subunit partitions with CaV2.1 into lipid rafts in cerebellum: implications for localization and function. J. Neurosci. 26, 8748–8757. doi: 10.1523/JNEUROSCI.2764-06.2006, 16928863 PMC6674382

[ref10] Debanne D. Gähwiler B. H. Thompson S. M. (1998). Long-term synaptic plasticity between pairs of individual CA3 pyramidal cells in rat hippocampal slice cultures. J. Physiol. 507, 237–247. doi: 10.1111/j.1469-7793.1998.237bu.x, 9490845 PMC2230782

[ref11] Eckert G. P. (2010). Manipulation of lipid rafts in neuronal cells. Open Biol. J. 3, 32–38. doi: 10.2174/18741967010030100032

[ref12] Edelmann E. Cepeda-Prado E. Franck M. Lichtenecker P. Brigadski T. Leßmann V. (2015). Theta burst firing recruits BDNF release and signaling in postsynaptic CA1 neurons in spike-timing-dependent LTP. Neuron 86, 1041–1054. doi: 10.1016/j.neuron.2015.04.007 25959732

[ref13] Edelmann E. Lessmann V. Brigadski T. (2014). Pre and post-synaptic twists in BDNF secretion and action in synaptic plasticity. Neuropharmacology 76, 610–627. doi: 10.1016/j.neuropharm.2013.05.043 23791959

[ref14] Falcón-Moya R. Pérez-Rodríguez M. Prius-Mengual J. Andrade-Talavera Y. Arroyo-García L. E. Pérez-Artés R. . (2020). Astrocyte-mediated switch in spike timing-dependent plasticity during hippocampal development. Nat. Commun. 11:4388. doi: 10.1038/s41467-020-18024-4, 32873805 PMC7463247

[ref15] Frank C. Rufini S. Tancredi V. Forcina R. Grossi D. D’Arcangelo G. (2008). Cholesterol depletion inhibits synaptic transmission and synaptic plasticity in rat hippocampus. Exp. Neurol. 212, 407–414. doi: 10.1016/j.expneurol.2008.04.019, 18559278

[ref16] Gottmann K. Mittmann T. Lessmann V. (2009). BDNF signaling in the formation, maturation and plasticity of glutamatergic and GABAergic synapses. Exp. Brain Res. 199, 203–234. doi: 10.1007/s00221-009-1994-z, 19777221

[ref17] Harward S. C. Hedrick N. G. Hall C. E. Parra-Bueno P. Milner T. A. Pan E. . (2016). Autocrine BDNF-TrkB signalling within a single dendritic spine. Nature 538, 99–103. doi: 10.1038/nature19766, 27680698 PMC5398094

[ref18] Humeau Y. Herry C. Kemp N. Shaban H. Fourcaudot E. Bissière S. . (2005). Dendritic spine heterogeneity determines afferent-specific Hebbian plasticity in the amygdala. Neuron 45, 119–131. doi: 10.1016/j.neuron.2004.12.019, 15629707

[ref19] Humeau Y. Reisel D. Johnson A. W. Borchardt T. Jensen V. Gebhardt C. . (2007). A pathway-specific function for different AMPA receptor subunits in amygdala long-term potentiation and fear conditioning. J. Neurosci. 27, 10947–10956. doi: 10.1523/jneurosci.2603-07.2007, 17928436 PMC6672841

[ref20] Incontro S. Musella M. L. Sammari M. Di Scala C. Fantini J. Debanne D. (2025). Lipids shape brain function through ion channel and receptor modulations: physiological mechanisms and clinical perspectives. Physiol. Rev. 105, 137–207. doi: 10.1152/physrev.00004.2024, 38990068

[ref21] Jung S. Y. Kim J. Kwon O. B. Jung J. H. An K. Jeong A. Y. . (2010). Input-specific synaptic plasticity in the amygdala is regulated by neuroligin-1 via postsynaptic NMDA receptors. Proc. Natl. Acad. Sci. USA 107, 4710–4715. doi: 10.1073/pnas.1001084107, 20176955 PMC2842073

[ref22] Karnovsky M. J. Kleinfeld A. M. Hoover R. L. Klausner R. D. (1982). The concept of lipid domains in membranes. J. Cell Biol. 94, 1–6. doi: 10.1083/jcb.94.1.1, 6889603 PMC2112185

[ref23] Korinek M. Gonzalez-Gonzalez I. M. Smejkalova T. Hajdukovic D. Skrenkova K. Krusek J. . (2020). Cholesterol modulates presynaptic and postsynaptic properties of excitatory synaptic transmission. Sci. Rep. 10:12651. doi: 10.1038/s41598-020-69454-5, 32724221 PMC7387334

[ref24] Korinek M. Vyklicky V. Borovska J. Lichnerova K. Kaniakova M. Krausova B. . (2015). Cholesterol modulates open probability and desensitization of NMDA receptors. J. Physiol. 593, 2279–2293. doi: 10.1113/jphysiol.2014.288209, 25651798 PMC4457192

[ref25] Koudinov A. R. Koudinova N. V. (2001). Essential role for cholesterol in synaptic plasticity and neuronal degeneration. FASEB J. 15, 1858–1860. doi: 10.1096/fj.00-0815fje, 11481254

[ref26] Maggo S. Ashton J. C. (2014). Effects of HMG-CoA reductase inhibitors on learning and memory in the guinea pig. Eur. J. Pharmacol. 723, 294–304. doi: 10.1016/j.ejphar.2013.11.018, 24296319

[ref27] Markram H. Lübke J. Frotscher M. Sakmann B. (1997). Regulation of synaptic efficacy by coincidence of postsynaptic APs and EPSPs. Science 275, 213–215. doi: 10.1126/science.275.5297.213 8985014

[ref28] Martínez-Gallego I. Pérez-Rodríguez M. Coatl-Cuaya H. Flores G. Rodríguez-Moreno A. (2022). Adenosine and astrocytes determine the developmental dynamics of spike timing-dependent plasticity in the somatosensory cortex. J. Neurosci. 42, 6038–6052. doi: 10.1523/JNEUROSCI.0115-22.2022, 35768208 PMC9351642

[ref29] Meis S. Bergado-Acosta J. R. Yanagawa Y. Obata K. Stork O. Munsch T. (2008). Identification of a neuropeptide S responsive circuitry shaping amygdala activity via the endopiriform nucleus. PLoS One 3:e2695. doi: 10.1371/journal.pone.0002695, 18628994 PMC2442874

[ref30] Meis S. Endres T. Lessmann V. (2012). Postsynaptic BDNF signalling regulates long-term potentiation at thalamo-amygdala afferents. J. Physiol. 590, 193–208. doi: 10.1113/jphysiol.2011.220434, 22083603 PMC3300056

[ref31] Meis S. Endres T. Lessmann V. (2020). Neurotrophin signalling in amygdala-dependent cued fear learning. Cell Tissue Res. 382, 161–172. doi: 10.1007/s00441-020-03260-3, 32845430 PMC7529623

[ref32] Minichiello L. (2009). TrkB signalling pathways in LTP and learning. Nat. Rev. Neurosci. 10, 850–860. doi: 10.1038/nrn2738, 19927149

[ref33] Mohajerani M. H. Sivakumaran S. Zacchi P. Aguilera P. Cherubini E. (2007). Correlated network activity enhances synaptic efficacy via BDNF and the ERK pathway at immature CA3 CA1 connections in the hippocampus. Proc. Natl. Acad. Sci. USA 104, 13176–13181. doi: 10.1073/pnas.0704533104, 17656555 PMC1941828

[ref34] Musumeci G. Sciarretta C. Rodriguez-Moreno A. Al Banchaabouchi M. Negrete-Diaz V. Costanzi M. . (2009). TrkB modulates fear learning and amygdalar synaptic plasticity by specific docking sites. J. Neurosci. 29, 10131–10143. doi: 10.1523/JNEUROSCI.1707-09.2009, 19675247 PMC6664965

[ref35] Nagappan G. Lu B. (2005). Activity-dependent modulation of the BDNF receptor TrkB: mechanisms and implications. Trends Neurosci. 28, 464–471. doi: 10.1016/j.tins.2005.07.003, 16040136

[ref36] Nevian T. Sakmann B. (2006). Spine Ca2+ signaling in spike-timing dependent plasticity. J. Neurosci. 26, 11001–11013. doi: 10.1523/jneurosci.1749-06.2006, 17065442 PMC6674669

[ref37] Nikolenko V. N. Oganesyan M. V. Rizaeva N. A. Kudryashova V. A. Nikitina A. T. Pavliv M. P. . (2020). Amygdala: neuroanatomical and morphophysiological features in terms of neurological and neurodegenerative diseases. Brain Sci. 10:502. doi: 10.3390/brainsci10080502, 32751957 PMC7465610

[ref38] Panja D. Bramham C. R. (2014). BDNF mechanisms in late LTP formation: a synthesis and breakdown. Neuropharmacology 76, 664–676. doi: 10.1016/j.neuropharm.2013.06.024 23831365

[ref39] Pape H. C. Paré D. (2010). Plastic synaptic networks of the amygdala for the acquisition, expression, and extinction of conditioned fear. Physiol. Rev. 90, 419–463. doi: 10.1152/physrev.00037.2009, 20393190 PMC2856122

[ref40] Park H. Poo M. M. (2013). Neurotrophin regulation of neural circuit development and function. Nat. Rev. Neurosci. 14, 7–23. doi: 10.1038/nrn3379, 23254191

[ref41] Pereira D. B. Chao M. V. (2007). The tyrosine kinase Fyn determines the localization of TrkB receptors in lipid rafts. J. Neurosci. 27, 4859–4869. doi: 10.1523/JNEUROSCI.4587-06.2007, 17475794 PMC6672086

[ref42] Pike F. G. Meredith R. M. Olding A. W. Paulsen O. (1999). Rapid report: postsynaptic bursting is essential for “Hebbian” induction of associative long-term potentiation at excitatory synapses in rat hippocampus. J. Physiol. 518, 571–576. doi: 10.1111/j.1469-7793.1999.0571p.x 10381601 PMC2269446

[ref43] Rodríguez-Moreno A. González-Rueda A. Banerjee A. Upton A. L. Craig M. T. Paulsen O. (2013). Presynaptic self-depression at developing neocortical synapses. Neuron 77, 35–42. doi: 10.1016/j.neuron.2012.10.035, 23312514 PMC3542421

[ref44] Rodríguez-Moreno A. Kohl M. M. Reeve J. E. Eaton T. R. Collins H. A. Anderson H. L. . (2011). Presynaptic induction and expression of timing-dependent long-term depression demonstrated by compartment-specific photorelease of a use-dependent NMDA receptor antagonist. J. Neurosci. 31, 8564–8569. doi: 10.1523/JNEUROSCI.0274-11.2011, 21653860 PMC4299820

[ref45] Rodrıguez-Moreno A. Paulsen O. (2008). Spike timing-dependent long-term depression requires presynaptic NMDA receptors. Nat. Neurosci. 11, 744–745. doi: 10.1038/nn.2125, 18516036

[ref46] Rumpel S. LeDoux J. Zador A. Malinow R. (2005). Postsynaptic receptor trafficking underlying a form of associative learning. Science 308, 83–88. doi: 10.1126/science.1103944, 15746389

[ref47] Schreurs B. G. (2010). The effects of cholesterol on learning and memory. Neurosci. Biobehav. Rev. 34, 1366–1379. doi: 10.1016/j.neubiorev.2010.04.010, 20470821 PMC2900496

[ref48] Shelton D. L. Sutherland J. Gripp J. Camerato T. Armanini M. P. Phillips H. S. . (1995). Human trks: molecular cloning, tissue distribution, and expression of extracellular domain immunoadhesins. J. Neurosci. 15, 477–491. doi: 10.1523/JNEUROSCI.15-01-00477.1995, 7823156 PMC6578290

[ref49] Shin R. M. Tsvetkov E. Bolshakov V. Y. (2006). Spatiotemporal asymmetry of associative synaptic plasticity in fear conditioning pathways. Neuron 52, 883–896. doi: 10.1016/j.neuron.2006.10.010, 17145508 PMC1764975

[ref50] Sigurdsson T. Doyère V. Cain C. K. LeDoux J. E. (2007). Long-term potentiation in the amygdala: a cellular mechanism of fear learning and memory. Neuropharmacology 52, 215–227. doi: 10.1016/j.neuropharm.2006.06.022, 16919687

[ref51] Simons K. Toomre D. (2000). Lipid rafts and signal transduction. Nat. Rev. Mol. Cell Biol. 1, 31–39. doi: 10.1038/35036052, 11413487

[ref52] Sjöstrom P. J. Rancz E. A. Roth A. Hausser M. (2008). Dendritic excitability and synaptic plasticity. Physiol. Rev. 88, 769–840. doi: 10.1152/physrev.00016.2007, 18391179

[ref53] Suzuki S. Numakawa T. Shimazu K. Koshimizu H. Hara T. Hatanaka H. . (2004). BDNF-induced recruitment of TrkB receptor into neuronal lipid rafts: roles in synaptic modulation. J. Cell Biol. 167, 1205–1215. doi: 10.1083/jcb.200404106, 15596541 PMC2172613

[ref54] Walz C. Jüngling K. Lessmann V. Gottmann K. (2006). Presynaptic plasticity in an immature neocortical network requires NMDA receptor activation and BDNF release. J. Neurophysiol. 96, 3512–3516. doi: 10.1152/jn.00018.2006, 17110740

[ref55] Woodson W. Farb C. R. Ledoux J. E. (2000). Afferents from the auditory thalamus synapse on inhibitory interneurons in the lateral nucleus of the amygdala. Synapse 38, 124–137. doi: 10.1002/1098-2396(200011)38:2<124::AID-SYN3>3.0.CO;2-N, 11018786

[ref56] Yamada D. Wada K. Sekiguchi M. (2016). Modulation of long-term potentiation of cortico-amygdala synaptic responses and auditory fear memory by dietary polyunsaturated fatty acid. Front. Behav. Neurosci. 10:164. doi: 10.3389/fnbeh.2016.00164, 27601985 PMC4993868

[ref57] Yoshii A. Constantine-Paton M. (2010). Postsynaptic BDNF-TrkB signaling in synapse maturation, plasticity, and disease. Dev. Neurobiol. 70, 304–322. doi: 10.1002/dneu.20765, 20186705 PMC2923204

[ref58] Zhang T. Yin Y. Xia X. Que X. Liu X. Zhao G. . (2026). Regulation of synaptic function and lipid metabolism. Neural Regen. Res. 21, 1037–1057. doi: 10.4103/NRR.NRR-D-24-01412, 40313084 PMC12296456

[ref59] Zonta B. Minichiello L. (2013). Synaptic membrane rafts: traffic lights for local neurotrophin signaling? Front. Synaptic Neurosci. 5:9. doi: 10.3389/fnsyn.2013.00009, 24151466 PMC3798807

